# Supplementary material for: Long Covid stigma: Estimating burden and validating scale in a UK-based sample
Source: PLoS One. 2022 Nov 23;17(11):e0277317. doi: 10.1371/journal.pone.0277317 (PMC9683629; doi:10.1371/journal.pone.0277317)
Supplement: S1 Table — (DOCX) [file pone.0277317.s001.docx]

Supplementary Table 1: Sample characteristics (n=1166)

|  | n | % |
| --- | --- | --- |
| **Age (mean 47.7, SD 10.6)** |  |  |
| 18-30 | 63 | 5.5 |
| 31-45 | 415 | 36.0 |
| 46-59 | 519 | 45.1 |
| ≥60 | 155 | 13.5 |
| Missing | 14 | 1.2 |
| **Gender** |  |  |
| Male | 173 | 15.0 |
| Female | 965 | 83.8 |
| Non-binary or other | 14 | 1.2 |
| Missing | 14 | 1.2 |
| **Ethnicity** |  |  |
| White | 1096 | 95.4 |
| Minority ethnic groups | 53 | 4.6 |
| Missing | 17 | 1.5 |
| **Country of residence** |  |  |
| UK – England | 778 | 67.3 |
| UK – Scotland | 111 | 9.6 |
| UK – Wales | 58 | 5.0 |
| UK – Northern Ireland | 9 | 0.8 |
| Outside the UK | 200 | 17.3 |
| Missing | 10 | 0.9 |
| **Educational** **qualification** |  |  |
| No formal qualifications | 13 | 1.1 |
| O levels or equivalent | 97 | 8.3 |
| A levels or equivalent | 151 | 13.0 |
| University degree or above | 902 | 77.5 |
| Other | 1 | 0.1 |
| Missing | 2 | 0.2 |
| **Duration of illness** |  |  |
| <12 months | 40 | 3.5 |
| 12-<15 months | 66 | 5.8 |
| 15-<18 months | 51 | 4.5 |
| >18 months | 989 | 86.3 |
| Missing | 20 | 1.7 |
| **Employment status** |  |  |
| Employed | 780 | 67.0 |
| Unable to work | 341 | 29 |
| Student/Volunteer | 25 | 2.2 |
| Unemployed and looking for work | 19 | 1.6 |
| Missing | 1 | 0.1 |
| **Clinical diagnosis of Long Covid received or on health record** |  |  |
| No | 53 | 4.8 |
| Not sure | 129 | 11.7 |
| Have test confirmation of initial Covid infection but no/not sure clinical diagnosis of Long Covid | 73 | 6.6 |
| No official diagnosis but doctors suspect I have Long Covid | 308 | 27.9 |
| Yes, Long Covid as a diagnosis on health record | 540 | 49.0 |
| Missing | 63 | 5.4 |
| **Long Covid Stigma Scale (LCSS) score**, mean (SD) | 20.5 ± 10.7 | |
| Missing | 99 | 8.5 |
| **Disclosure concerns**, mean (SD) | 2.9 ± 2.3 | |
| Missing | 71 | 6.1 |
| **PHQ-8 score**, mean (SD) | 9.2 ± 5.8 | |
| Missing | 103 | 8.8 |
