# Supplementary material for: Long Covid stigma: Estimating burden and validating scale in a UK-based sample
Source: PLoS One. 2022 Nov 23;17(11):e0277317. doi: 10.1371/journal.pone.0277317 (PMC9683629; doi:10.1371/journal.pone.0277317)
Supplement: S2 Table — (DOCX) [file pone.0277317.s002.docx]

Supplementary Table 2: Response option frequencies for each stigma item

|  | Full sample  (n=1067) | | | | | Clinical diagnosis  (n=516) | | | | | No clinical diagnosis/unsure  (n=543) | | | | |
| --- | --- | --- | --- | --- | --- | --- | --- | --- | --- | --- | --- | --- | --- | --- | --- |
| Response options* | 0 | 1 | 2 | 3 | 4 | 0 | 1 | 2 | 3 | 4 | 0 | 1 | 2 | 3 | 4 |
| **Enacted stigma items** |  | | | | |  | | | | |  | | | | |
| Because of my illness, some people seemed uncomfortable with me | 27.5 | 20.4 | 37.6 | 13.3 | 1.3 | 18.8 | 20.5 | 44.3 | 14.5 | 1.9 | 35.4 | 20.2 | 31.8 | 11.9 | 0.7 |
| Because of my illness, some people were unkind to me | 48.2 | 21.8 | 22.8 | 7.0 | 0.3 | 40.7 | 24.0 | 27.4 | 7.7 | 0.2 | 55.2 | 19.6 | 18.7 | 6.2 | 0.4 |
| People I care about stopped contacting me after learning I have Long Covid | 54.5 | 17.1 | 19.3 | 8.4 | 0.8 | 43.4 | 19.4 | 25.4 | 11.2 | 0.6 | 64.9 | 15.0 | 13.4 | 5.6 | 1.1 |
| People have acted as if I am dishonest since I have had Long Covid | 45.6 | 20.2 | 23.3 | 9.3 | 1.6 | 42.1 | 22.5 | 23.6 | 10.9 | 0.9 | 48.9 | 17.9 | 23.3 | 7.8 | 2.2 |
| I have been treated with less respect than other people are because of Long Covid | 48.1 | 20.0 | 21.5 | 9.6 | 0.8 | 40.0 | 22.3 | 24.4 | 12.2 | 1.1 | 55.3 | 18.1 | 18.8 | 7.2 | 0.5 |
| **Internalised stigma items** |  |  |  |  |  |  |  |  |  |  |  |  |  |  |  |
| I have felt embarrassed about my illness | 27.8 | 12.1 | 30.5 | 21.4 | 8.2 | 21.9 | 12.2 | 32.0 | 24.1 | 9.9 | 32.7 | 12.3 | 29.6 | 19.0 | 6.5 |
| I have felt embarrassed because of my physical limitations | 16.1 | 9.4 | 29.5 | 31.3 | 13.8 | 11.1 | 8.1 | 28.0 | 36.3 | 16.5 | 20.0 | 10.8 | 31.2 | 27.0 | 11.0 |
| I feel that I have been tainted by Long Covid and am of less value than others because of it | 27.1 | 16.3 | 26.7 | 19.1 | 10.8 | 18.4 | 15.0 | 28.0 | 23.8 | 14.8 | 34.8 | 17.8 | 25.8 | 15.0 | 6.7 |
| I have felt like I am very different from other people on account of Long Covid | 17.5 | 14.9 | 32.2 | 21.9 | 13.5 | 11.3 | 12.8 | 31.4 | 27.6 | 16.9 | 23.1 | 16.8 | 33.0 | 16.8 | 10.3 |
| **Anticipated stigma items** |  |  |  |  |  |  |  |  |  |  |  |  |  |  |  |
| Many people tend to think Long Covid isn’t a real illness | 7.6 | 12.8 | 36.2 | 33.5 | 10.0 | 6.9 | 13.6 | 37.6 | 31.0 | 10.8 | 7.9 | 12.1 | 34.7 | 35.9 | 9.4 |
| I feel that some people assume that having Long Covid is a sign of personal weakness | 18.1 | 15.9 | 34.6 | 23.9 | 7.6 | 12.8 | 15.0 | 36.7 | 26.3 | 9.2 | 23.0 | 16.6 | 32.4 | 21.9 | 6.2 |
| I worry that people with Long Covid lose their jobs when their employers find out | 19.3 | 14.6 | 36.5 | 22.1 | 7.5 | 13.2 | 13.5 | 37.4 | 26.9 | 9.0 | 24.6 | 15.7 | 36.2 | 17.5 | 6.0 |
| I worry that people may judge me negatively when they learn I have Long Covid | 21.4 | 17.6 | 32.8 | 19.5 | 8.7 | 13.8 | 16.8 | 34.8 | 23.0 | 11.6 | 28.4 | 18.4 | 31.1 | 16.1 | 6.0 |
| *Response options indicate 0:Never; 1:Rarely; 2:Sometimes; 3:Often; 4:Always | | | | | | | | | | | | | | | |
