# Supplementary material for: Long Covid stigma: Estimating burden and validating scale in a UK-based sample
Source: PLoS One. 2022 Nov 23;17(11):e0277317. doi: 10.1371/journal.pone.0277317 (PMC9683629; doi:10.1371/journal.pone.0277317)
Supplement: S3 Table — (DOCX) [file pone.0277317.s003.docx]

Supplementary Table 3: Factor loadings of individual stigma items on subscales of internalised, enacted and anticipated stigma using confirmatory factor analysis

|  | Full sample  (n=1067) | | | Clinical diagnosis  (n=516) | | | No clinical diagnosis/unsure  (n=543) | | |
| --- | --- | --- | --- | --- | --- | --- | --- | --- | --- |
|  | Enacted | Internalised | Anticipated | Enacted | Internalised | Anticipated | Enacted | Internalised | Anticipated |
| Because of my illness, some people seemed uncomfortable with me | 0.78 |  |  | 0.75 |  |  | 0.79 |  |  |
| Because of my illness, some people were unkind to me | 0.79 |  |  | 0.80 |  |  | 0.78 |  |  |
| People I care about stopped contacting me after learning I have Long Covid | 0.70 |  |  | 0.66 |  |  | 0.71 |  |  |
| People have acted as if I am dishonest since I have had Long Covid | 0.76 |  |  | 0.77 |  |  | 0.77 |  |  |
| I have been treated with less respect than other people are because of Long Covid | 0.86 |  |  | 0.86 |  |  | 0.86 |  |  |
| I have felt embarrassed about my illness |  | 0.76 |  |  | 0.74 |  |  | 0.76 |  |
| I have felt embarrassed because of my physical limitations |  | 0.75 |  |  | 0.72 |  |  | 0.77 |  |
| I feel that I have been tainted by Long Covid and am of less value than others because of it |  | 0.83 |  |  | 0.82 |  |  | 0.83 |  |
| I have felt like I am very different from other people on account of Long Covid |  | 0.73 |  |  | 0.67 |  |  | 0.76 |  |
| Many people tend to think Long Covid isn’t a real illness |  |  | 0.64 |  |  | 0.70 |  |  | 0.63 |
| I feel that some people assume that having Long Covid is a sign of personal weakness |  |  | 0.77 |  |  | 0.79 |  |  | 0.74 |
| I worry that people with Long Covid lose their jobs when their employers find out |  |  | 0.58 |  |  | 0.51 |  |  | 0.60 |
| I worry that people may judge me negatively when they learn I have Long Covid |  |  | 0.86 |  |  | 0.84 |  |  | 0.87 |
| CFI |  | 0.970 |  |  | 0.975 |  |  | 0.968 |  |
| TLI |  | 0.956 |  |  | 0.963 |  |  | 0.952 |  |
| RMSEA |  | 0.065 |  |  | 0.058 |  |  | 0.068 |  |
| SRMR |  | 0.037 |  |  | 0.040 |  |  | 0.039 |  |
| χ2/df |  | 5.5 |  |  | 2.7 |  |  | 3.5 |  |
| Cronbach’s alpha | 0.88 | 0.86 | 0.82 | 0.87 | 0.84 | 0.81 | 0.89 | 0.86 | 0.82 |
