# Supplementary material for: Long Covid stigma: Estimating burden and validating scale in a UK-based sample
Source: PLoS One. 2022 Nov 23;17(11):e0277317. doi: 10.1371/journal.pone.0277317 (PMC9683629; doi:10.1371/journal.pone.0277317)
Supplement: S4 Table — (DOCX) [file pone.0277317.s004.docx]

Supplementary Table 4: Correlations between stigma scores, eight-item Patient Health Questionnaire (PHQ-8 score) and disclosure concerns

|  | Full sample  (n=1067) | | | | Clinical diagnosis  (n=516) | | | | No clinical diagnosis/unsure  (n=543) | | | |
| --- | --- | --- | --- | --- | --- | --- | --- | --- | --- | --- | --- | --- |
|  | PHQ-8 score | p-value | Disclosure concerns | p-value | PHQ-8 score | p-value | Disclosure concerns | p-value | PHQ-8 score | p-value | Disclosure concerns | p-value* |
| Overall LCSS | 0.46 | <0.001 | 0.62 | <0.001 | 0.45 | <0.001 | 0.65 | <0.001 | 0.46 | <0.001 | 0.62 | <0.001 |
| Enacted stigma subscale | 0.34 | <0.001 | 0.50 | <0.001 | 0.31 | <0.001 | 0.53 | <0.001 | 0.35 | <0.001 | 0.48 | <0.001 |
| Internalised stigma subscale | 0.48 | <0.001 | 0.53 | <0.001 | 0.47 | <0.001 | 0.55 | <0.001 | 0.47 | <0.001 | 0.53 | <0.001 |
| Anticipated stigma subscale | 0.38 | <0.001 | 0.59 | <0.001 | 0.38 | <0.001 | 0.59 | <0.001 | 0.38 | <0.001 | 0.61 | <0.001 |

*Comparisons used Pearson’s correlation
