# Supplementary material for: Long Covid stigma: Estimating burden and validating scale in a UK-based sample
Source: PLoS One. 2022 Nov 23;17(11):e0277317. doi: 10.1371/journal.pone.0277317 (PMC9683629; doi:10.1371/journal.pone.0277317)
Supplement: S5 Table — (DOCX) [file pone.0277317.s005.docx]

Supplementary Table 5: Prevalence of reported stigma

|  | Experienced stigma sometimes or more often | | | | Experienced stigma often/always | | | |
| --- | --- | --- | --- | --- | --- | --- | --- | --- |
|  | Full sample (n=1067) | Clinical diagnosis  (n=516) | No clinical diagnosis/unsure  (n=543) | p-value | Full sample (n=1067) | Clinical diagnosis  (n=516) | No clinical diagnosis/unsure  (n=543) | p-value* |
| Overall LCSS | 95.1 | 97.5 | 93.0 | 0.001 | 76.2 | 83.0 | 70.2 | <0.001 |
| Enacted stigma | 63.4 | 71.3 | 56.6 | <0.001 | 25.6 | 29.1 | 22.3 | 0.01 |
| Internalised stigma | 86.8 | 92.2 | 82.1 | <0.001 | 59.3 | 69.1 | 50.4 | <0.001 |
| Anticipated stigma | 90.1 | 93.0 | 88.4 | 0.009 | 59.5 | 63.5 | 56.0 | 0.01 |

* Comparisons between those with a clinical diagnosis and those with no clinical diagnosis/unsure used chi square test.
