# Supplementary material for: Long Covid stigma: Estimating burden and validating scale in a UK-based sample
Source: PLoS One. 2022 Nov 23;17(11):e0277317. doi: 10.1371/journal.pone.0277317 (PMC9683629; doi:10.1371/journal.pone.0277317)
Supplement: Attachment — Submitted filename: Response-DataAvailability.docx [file pone.0277317.s006.docx]

1. We note your current Data Availability statement is:

- "No - some restrictions will apply"

- "The survey data is available on request provided ethics committee approval for sharing the anonymised data is granted. To request access conditional on approval, please email [rgoinfo@soton.ac.uk](mailto:rgoinfo@soton.ac.uk)."

In line with our goal of ensuring long-term data availability to all interested researchers, PLOS’ Data Policy states that authors cannot be the sole named individuals responsible for ensuring data access (<https://eur03.safelinks.protection.outlook.com/?url=http%3A%2F%2Fjournals.plos.org%2Fplosone%2Fs%2Fdata-availability%23loc-acceptable-data-sharing-methods&amp;data=05%7C01%7CNida.Ziauddeen%40soton.ac.uk%7Cae6e9580b68542d9aede08daa0628b60%7C4a5378f929f44d3ebe89669d03ada9d8%7C0%7C0%7C637998642339027662%7CUnknown%7CTWFpbGZsb3d8eyJWIjoiMC4wLjAwMDAiLCJQIjoiV2luMzIiLCJBTiI6Ik1haWwiLCJXVCI6Mn0%3D%7C3000%7C%7C%7C&amp;sdata=DyEmMuPidN2k3xuyQ5XlmvjPjTwi7L%2FnhqoPzGS7KXo%3D&amp;reserved=0>).

Please clarify who is the recipient of the provided contact email, "[rgoinfo@soton.ac.uk](mailto:rgoinfo@soton.ac.uk)" and what their role is as it relates to the relevant data.

*The provided contact email, "*[*rgoinfo@soton.ac.uk*](mailto:rgoinfo@soton.ac.uk)*" is the inbox of the University of Southampton's Research Integrity and Governance team (part of Research and Innovation Services). The team provides help and support to staff and students on matters related to research integrity, research ethics and research governance including clinical governance.*
